# Tracking the source of microplastics in soil—an exploratory case study in peach orchards from east-central Portugal

**DOI:** 10.1007/s10661-025-14072-9

**Published:** 2025-05-10

**Authors:** Abel Veloso, Vera Silva, Esperanza Huerta Lwanga, Nicolas Beriot, Maria do Carmo Horta, Maria Paula Simões, Violette Geissen

**Affiliations:** 1 Soil Physics and Land Management Group, Wageningen University and Research, Wageningen, The Netherlands; 2 School of Agriculture, Polytechnic Institute of Castelo Branco, Castelo Branco, Portugal; 3 Research Centre for Natural Resources, Environment and Society (CERNAS), Castelo Branco, Portugal

**Keywords:** Microplastic sources, Permanent crops, Agricultural soil, Irrigation water, Manure, Atmospheric deposition

## Abstract

In the last 20 years, world plastic production has increased rapidly, reaching 4.00 × 10^11^ kg in 2022. However, less than 10% was recycled. Moreover, most conventional plastics are persistent and, therefore, remain in the environment long after their release. Although most studies on microplastic contamination focus on a single environmental compartment, an integrated and multicompartment approach is highly recommended considering the multitude of interactions between those compartments. This study addresses this knowledge gap, investigating the presence and potential sources of microplastics (MP) in agricultural soils under typical conditions of the Mediterranean region, characterised by dry summers and relatively moist and mild winters (Csa according to the Köppen-Geiger classification). For this, 19 orchards from east-central Portugal were used as case study sites, and a total of 111 samples were analysed. Soil content in MP was assessed in 3 soil layers (0–5, 5–15 and 15–25 cm). To quantify potential sources to soil, information from farmers was used, and samples from irrigation water, atmospheric deposition and manure were analysed. Optical assessment with the assistance of a stereomicroscope was used for MP quantification. The 0–5 cm soil layer showed a lower content (average of 2.2 particles·g^−1^) and higher particle sizes (average of 168 µm) than deeper soil layers. The identified sources contributed with 1.02 × 10^5^ particles·m^−2^·year^−1^. Irrigation water was the main source, representing 55.9% of the aforementioned input rate encountered for the 3 identified MP sources. Additional knowledge is needed regarding the possible variation in MP content throughout the year and between years and the polymer identification, not only in samples collected from soil but also from its main contamination sources. Furthermore, this study should be extended to other crops and regions as part of broader soil health monitoring.

**Supplementary Information:**

The online version contains supplementary material available at 10.1007/s10661-025-14072-9.

## Introduction

Plastic materials are ubiquitous in our society. Since the onset of their large-scale production, in the mid-twentieth century, plastic manufacture has increased rapidly, reaching a total of 4.00 × 10^11^ kg in 2022. Considering that global plastic production in 2002 was 2.31 × 10^11^ kg, this corresponds to a 73% increase in just 2 decades (Plastics Europe, 2023; Richie et al., 2023). Furthermore, the production of plastic is projected to continue its rapid rise, reaching a global total of 1.2 × 10^12^ kg by 2060 (OECD, 2022). Correspondingly, plastic waste is expected to follow this trend, with less than 20% of plastic waste projected to be recycled by that year (OECD, 2022).

There remains a lack of comprehensive legislation addressing microplastic (MP) soil contamination within the European Union (EU) (European Commission, 2024b). Nonetheless, several initiatives are being adopted to tackle this issue. The Zero Pollution Action Plan aims to reduce MP pollution by 30% by 2030 compared to the current situation (European Commission, 2021, 2024b). In 2023, the EU imposed restrictions on the sale of MP and products containing MP under the REACH chemical legislation (European Commission, 2023). Additionally, there is a proposal for regulation to prevent the loss of plastic pellets (European Commission, 2024b). The EU strategy on plastics is guided by 4 main pillars (European Commission, 2024c): (1) making recycling more profitable; (2) reducing plastic waste; (3) developing plastic materials that are easier to recycle, enhancing the efficiency of the recycling process, and tracing and removing hazardous ingredients from recycled plastic; and (4) finding global solutions and setting global standards for plastics. Biodegradable, compostable, and biobased plastics are considered by the EU as possible alternatives to conventional fossil-based plastics. However, the EU policy framework also stresses that these alternatives must be approached with caution, as they present their own challenges (European Commission, 2024a).

Due to their very slow degradation, most conventional plastic materials tend to persist indefinitely in the environment, particularly in the form of MP (less than 5 mm) and nanoplastics (1 nm – 1 µm) (Beriot et al., 2021; Li et al., 2023; Meng et al., 2021). The high mobility of MP and nanoplastics accounts for their presence in remote locations, without obvious sources of plastic contamination (Beriot et al., 2021; He et al., 2018; Lwanga et al., 2022).

MP derived from fossil plastics can be highly persistent in soil, leading to their accumulation in this matrix (Barnes et al., 2009; De Souza Machado et al., 2018). Agricultural soils are no exception. There are several possible sources: plastic supplies used in agricultural activities; soil amendments such as sewage sludge, manure, or compost; and irrigation water and atmospheric deposition. The specific significance of each one is highly dependent on factors such as regional activities and edaphoclimatic conditions (Corradini et al., 2021; De Souza Machado et al., 2018; Lwanga et al., 2022).

Plastic supplies include irrigation pipes, mulch films, strings/ropes and protective nets (FAO, 2021; Lwanga et al., 2022). Polyethylene and polypropylene are the 2 most commonly used plastic polymers in agriculture. Unsurprisingly, these are amongst the most frequently found polymers in soil and other agriculture-related matrices, such as irrigation water (Jiang et al., 2023; Yang et al., 2021). To prevent their spread in the environment, plastic residues should be collected and stored in a dry, dark place protected from the wind before being sent for proper treatment. Nevertheless, even when plastic materials are used according to manufacturers’ and legal requirements, particles released through abrasion or photodegradation will occur. This is acknowledged, for example, by Regulation (EU) 2020/740 on the labelling of tires, which identifies that tire wear as a significant source of MP (European Commission, 2020).

In addition to the direct agricultural use of plastic supplies, which accounted for 3.4% of the 50.7 × 10^9^ kg of plastic used in Europe in 2019, organic soil amendments, including sewage sludge, manure, and compost, are other important sources of MP in soil (Lwanga et al., 2022). Wastewater treatment plants can be highly effective in removing MP from water, with removal rates ranging from 50% to over 99%, thereby trapping MP in the resulting sewage sludge (Corradini et al., 2019; Murphy et al., 2016; Sadia et al., 2022). Consequently, the application of sewage sludge to soil can be a significant source of plastic particles, as demonstrated by several studies (Corradini et al., 2019; Harley-Nyang et al., 2022; Ren et al., 2020). Manure and compost can also contribute MP to soil, as shown by Yang et al. (2021), who found a significantly higher MP content in soils where pig manure had been applied for 22 years compared to fields without manure application. Similarly, Lwanga et al. (2023) reported higher MP content in soils amended with compost compared with soils without compost application over the last 5 years. MP have also been detected in manure and compost themselves, ranging from 997 to 4520 particles·kg^−1^ (Beriot et al., 2021; Yang et al., 2021; J. Zhang et al., 2023).

Once in soil, plastic debris can be further transported by wind and water ending, in most cases, in the marine environment (Dahl et al., 2021; Lwanga et al., 2022; Segur & Sonke, 2024). Increased aridity due to climate change favours wind erosion and, consequently, MP transport (Lwanga et al., 2022; Rezaei et al., 2019). Several factors influence MP transport through air and their deposition. Smaller particles from light-density polymers are more readily transported, and their deposition is favoured by precipitation and, during dry periods, by higher relative humidity (Lwanga et al., 2023; Rezaei et al., 2022; Szewc et al., 2021).

MP can also migrate into deeper soil layers. This migration depends on factors such as MP sizes, shapes, materials, and the soil characteristics, particularly soil pore sizes and the presence of soil cracks (H. Zhang et al., 2022; Z. Zhao et al., 2022). Water infiltration, enhanced by irrigation, is a potential contributor to MP transport into deeper layers, as demonstrated by Zhao et al. (2022) and Tehrani et al. (2023). This effect was observed for various polymers, including polyethylene, polybutylene adipate terephthalate, and starch-based plastics, with especially high MP content found in soil cracks. Additionally, soil fauna, such as earthworms, can act as carriers of MP into deeper layers, either in their guts or attached to their skin (Lwanga et al., 2017; Rillig et al., 2017).

Once in soil, MP can change soil properties and interact, often negatively, with organisms that provide essential ecosystem services, including earthworms (De Souza Machado et al., 2018; Lwanga et al., 2017; Mongil-Manso et al., 2023; Nath et al., 2024). In addition to this, MP can adsorb and release harmful chemical and biological contaminants, such as additives used during the manufacture of the original plastic materials, antibiotic-resistant genes, RNA fragments, and microorganisms, some of which may be pathogenic (Maguire & Gardner, 2023). Smaller particles can also be absorbed by crops, thereby entering the food chain (De Souza Machado et al., 2018; Yu et al., 2024).

Despite the increasing attention that MP contamination has received in recent years, studies that identify and compare the main sources of MP in soil are still relatively rare. In this regard, Lwanga et al. (2023) evaluated, the content of MP not only in agricultural soils but also in nearby water bodies, airborne dust, and ditch sediments in samples collected in the Netherlands. A positive and significant correlation was found between the polypropylene content in soils and the polypropylene content in ditch sediments. Guo et al. (2023) identified organic fertilisers and irrigation water as the main sources of MP in soil in a study conducted in China, in agricultural facilities.

However, understanding the state of soil contamination and identifying and characterising its main sources, although highly dependent on factors such as edaphoclimatic conditions and local activities, are of great importance, if not to eliminate, at least to reduce soil contamination by plastics. This study addresses this knowledge gap, using peach orchards from the east-central Portuguese region of Beira Interior as case study sites. Despite the high importance of this crop in the local economy, to our knowledge, the MP contamination in soil under peach orchards from this region has never been addressed. However, like other crops, orchards rely heavily in irrigation systems made almost entirely of plastic. Considering that a significant portion of this irrigation system is above ground and the general sensitivity of plastic materials to light, it is predictable that irrigation water is an important source of MP in soil, especially in southern Europe where sunshine duration is high.

Several studies have highlighted not only the risks of the soil MP contamination but also the possible, and usually harmful, interactions of MP with other contaminants, such as pesticide residues, which are intensively used in peach orchards and in other perennial crops (Hu et al., 2023; Maguire & Gardner, 2023; Tona et al., 2018; C. Wu et al., 2022). Therefore, the main objective of this study was to determine MP content in topsoil layer (0–5 cm) under peach orchards located in the east-central Portuguese region of Beira Interior. Additionally, we aimed to evaluate the MP content in 2 deeper soil layers (5–15 cm and 15–25 cm) and to identify and characterise the major potential sources of MP contamination in those orchards.

## Materials and methods

### Site characterisation

**Fig. 1:**
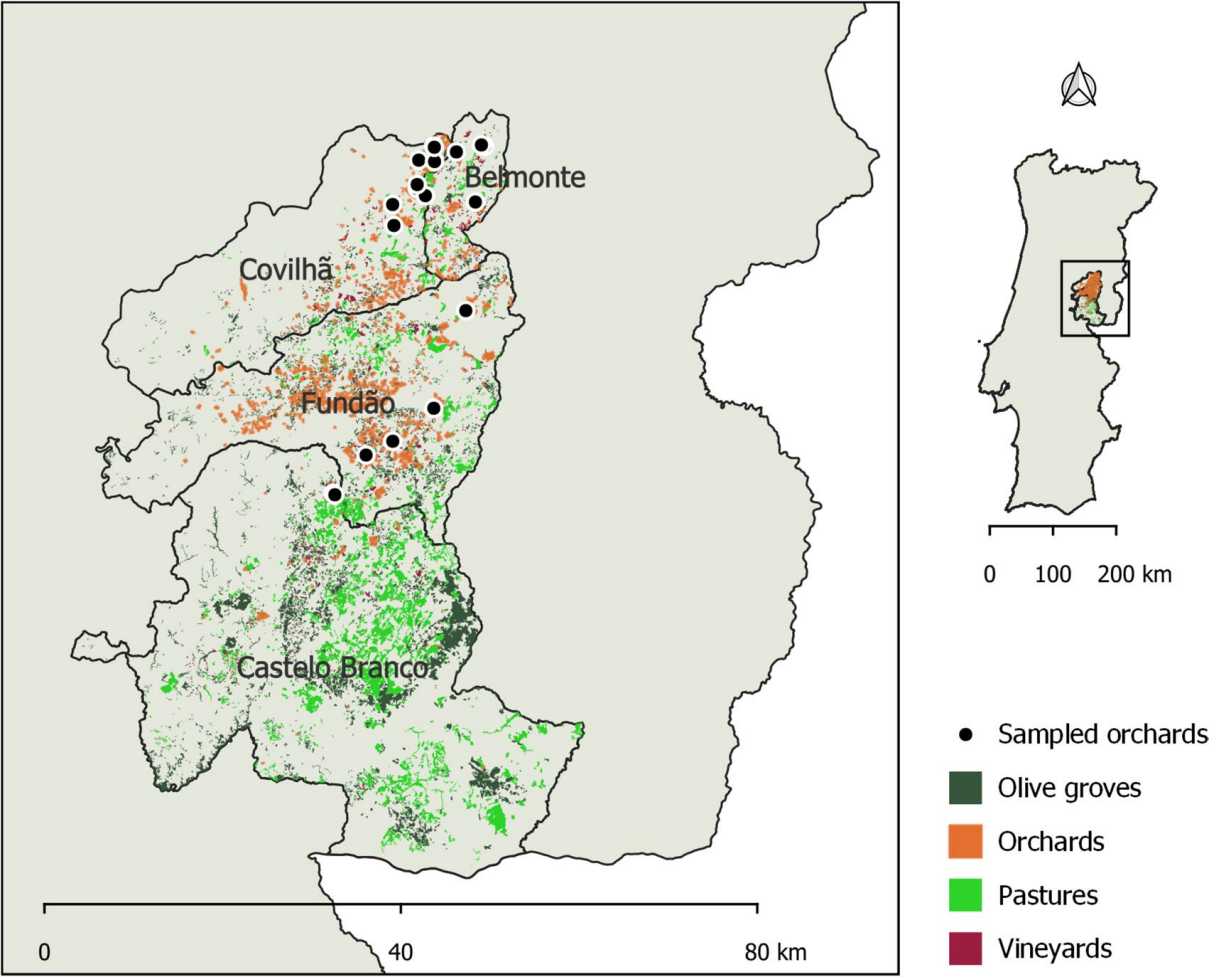
Distribution of the 19 peach orchards and the main crops (olive groves, orchards, pastures and vineyards) in the municipalities of Castelo Branco, Fundão, Covilhã, and Belmonte located in the east-central Portuguese region of Beira Interior. The data regarding the distribution of the main crops in the 4 municipalities that are shown in the figure was compiled with QGIS (version 3.28) and can be assessed at DGT (n.d.)

The orchards are located in the municipalities of Castelo Branco, Fundão, Covilhã, and Belmonte, in the east-central Portuguese region of Beira Interior (Fig. 1). The region experiences a hot summer Mediterranean climate (Csa according to the Köppen-Geiger classification) and the soils are predominantly Dystric Cambisols, derived from granite or schist, with a coarse texture and moderately acidic pH (FAO, 2015; Kottek et al., 2006). Olive groves, orchards, pastures, and vineyards are the main crops in this region. Amongst the orchards, almond, peach, sweet cherry, and apple are the most representative crops. Notably, this region has the country’s largest area of peach and sweet cherry production, representing 48% and 47% of the country’s total area, respectively (Instituto Nacional de Estatística, 2019).

A total of 19 peach orchards are included in this study. The typical tree density is approximately 550 trees.ha^−1^. All orchards are managed under Integrated Production rules, and their areas range between 0.47 ha and 4.50 ha (average of 1.99 ha). The exact locations of the orchards are not provided to protect farmers’ anonymity.

The orchards in this region are managed under similar conditions. More precisely, after the installation of the orchard, the soil is never tilled. Instead, weeds in the tree rows are controlled using periodical herbicide applications, whilst weeds in the inter-rows are mowed periodically. The irrigation of the orchards is conducted using a drip system typically made of polyethylene. Drip lines are located above ground, either directly on the soil surface or elevated by approximately 40–50 cm. The remaining irrigation pipes are mostly underground. The water used for irrigation is sourced either from rainfed ponds within the orchard or from dams. In the latter case, the water is delivered to the orchard through a pipeline that serves multiple farmers. Manure, primarily from sheep, is applied typically every 2 years. Sewage sludge was not applied in any of the orchards during at least the 2 years prior to sampling.

### Sampling

For this study, soil, water, atmospheric deposition, and manure samples were collected from some or all of the 19 peach orchards (Table 1). Topsoil samples (0–5 cm) constituted the main focus of our study and, therefore, were collected in all of the 19 orchards. The other groups of samples were collected only in some of these. Whilst financial and time constraints influenced this decision, there were also other and more specific reasons. Soil samples from deeper layers (5–15 cm and 15–25 cm) were collected in 9 orchards, where the compactness of the soil and its resistance were sufficiently low to allow the use of the soil auger at those depths. Water samples were collected in the orchards, 4 in total, where the irrigation schedule was made available by the farmers. Atmospheric deposition samples were collected in 5 orchards. Time and financial constraints, regarding not only the sample collection but also the quantification, were the main reasons for this. Manure samples were collected in 3 orchards, where we received the information from the farmers regarding the application of this soil amendment.

**Table 1:** Correspondence between the types of samples and the peach orchards from the east-central Portuguese region of Beira Interior, where they were collected. To protect farmers’ anonymity, orchards are designated by their codes

Orchard	Soil (0–5 cm)	Soil (5–15 cm and 15–25 cm)	Water (drippers and deposits)	Atmospheric deposition	Manure
F01	x		x	x	x
F02	x	x			
F03	x				
F04	x		x	x	x
F05	x	x			
F06	x		x	x	
F07	x				
F08	x	x			
F09	x	x			
F10	x	x		x	x
F11	x	x			
F12	x	x			
F13	x	x			
F14	x				
F15	x				
F16	x				
F17	x				
F18	x	x		x	
F19	x		x		

#### Soil samples

Soil samples were collected in October 2022, from all 19 peach orchards. The sampling procedure was adapted from the Land Use and Coverage Area frame Survey (LUCAS) and the European Food Safety Agency (EFSA) guidelines for in-row and permanent crops. Three composite samples of approximately 1 kg each were collected per orchard at a depth of 0–5 cm. Each composite sample was formed by combining 5 sub-samples collected within a tree row at equal distances. Sampling was performed with a metal soil auger, and all samples were stored in paper bags to minimise plastic contamination. After collection, samples were air-dried at room temperature and sieved (2 mm).

To evaluate whether the MP content of deeper soil layers is similar to that of the surface layer, 2 additional sets of composite samples were collected simultaneously at depths of 5–15 cm and 15–25 cm. Due to logistical reasons, these 2 groups of samples were collected in a subset of 9 orchards (Table 1). The sampling procedure followed the same method described for samples collected at 0–5 cm.

#### Water samples

In 4 orchards, 2 groups of water samples were collected in July 2022: from the drippers and from the water deposit located within the farm. Each sample had a volume of 1 L, similar to the method described by Haque et al. (2024). To increase the representativeness, each dripper sample was collected below the drippers at several points within the orchard.

These samples were used to (1) evaluate the contribution of irrigation water to MP contamination in soil and (2) determine whether the irrigation pipelines are a significant source of MP in the irrigation water and, consequently, in the soil. To minimise contamination, the samples were collected and stored in 1 L glass flasks until analysis.

#### Manure samples

Samples of sheep manure were collected in August 2023 at a rate of 1 composite sample per orchard from 3 orchards where manure was available (Table 1). Samples were stored in paper bags to minimise contamination.

#### Atmospheric deposition samples

Atmospheric deposition samples were collected in August 2023, from 5 orchards by placing 1 L glass flasks (7.4-cm diameter) without lids on the soil surface (Table 1) at a rate of 3 samples/flasks per orchard. Each flask was placed within a tree row and left open for 12 days. After this period, the flasks were collected, closed with their lids, and stored until analysis. There was no precipitation during the sampling period.

### Microplastic extraction

#### Soil samples

The procedure for extraction and identification of MP was based on Zhang et al. (2018) and on Beriot et al. (2021). Briefly, 1.0 g of soil was weighed into 50 mL tubes, and 10 mL of deionised water was added. The mixture was homogenized in a vortex for 10 s, shaken at 180 rpm for 15 min, and then centrifuged at 2500 rpm for 15 min. The supernatant was filtered using a vacuum apparatus and a Whatman paper filter (8 µm pore size). The extraction procedure was performed 3 times per sample. Each filter was stored inside a glass Petri dish and dried overnight in an oven at 35 °C.

#### Water samples

The procedure was adapted from Lwanga et al. (2023). Briefly, the water sample inside the glass flask was homogenized for 1 min with a metal spoon. Immediately after, 10 mL of water was collected and filtered using a filtering apparatus similar to what was described for soil samples. All handling materials were thoroughly washed with deionised water to recover as many plastic particles as possible. The filters were then placed in glass Petri dishes and dried overnight in an oven at 35 °C.

#### Atmospheric deposition samples

The interior of the flasks was thoroughly washed with deionised water using a filtering apparatus similar to what was described for soil samples. The filters were placed in glass Petri dishes to avoid contamination and dried overnight in an oven at 35 °C.

#### Manure samples

Due to the high percentage of organic matter, a digestion step was included for the manure samples. Specifically, 1.0 g of manure was weighed to 50 mL tubes, and 20 mL of 33% (w/w) hydrogen peroxide was added. The samples were left for 1 week at room temperature with their lids loose but still on top of the tubes to minimise contamination and allow gases from the decomposition of organic matter and hydrogen peroxide to escape. After this period, the extraction procedure was similar to that described for soil samples. The filters were placed in glass Petri dishes to avoid contamination and dried overnight in an oven at 35 °C.

### Microplastic detection and measurements

The particles present on each filter were carefully transferred to a glass slide and observed under a stereomicroscope (Leica S APO) equipped with a camera (Leica MC 190 HD). Two sets of photographs were taken at 10 × magnification: one before and one after heating the slide for 30 s at 150 °C on a hot plate.

The two sets of photographs were compared, and MP particles were identified based on colour, shape, brightness and increase in shine and circularity in response to heat (Beriot et al., 2021; S. Zhang et al., 2018). The number of MP and their area were determined from the photographs taken before heating, using Gimp (version 2.10) and ImageJ (version 1.53 t) software. The length of each particle was estimated by calculating the square root of its projected surface area.

### Method validation and quality control

Possible lab contamination and cross-contamination was evaluated by including 1 blank sample per batch. Considering the difficulty in finding field samples free from MP contamination and considering the need to evaluate possible contaminations during the analysis, distilled water was used for the blank samples. However, to increase the representativeness of the method validation applied to soil samples, soil heated at 450 °C for 16 h was used to eliminate any existing MP contamination.

The number of plastic particles identified in the blank samples varied between 0 and 3 (average of 0.52). The methods used for the extraction of soil/manure, water, and air deposition samples were validated by intentionally contaminating a group of samples from each type with a known content of yellow polyethylene particles ground from an empty and washed container and sieved at < 500 µm. Their extraction followed the aforementioned procedures.

#### Soil samples

Due to the high number of soil samples that were processed, 2 recovery/validation tests were performed for this type of samples. The recovery rate of the method was evaluated using 5 and 4 intentionally contaminated samples for the 1 st and the 2nd recovery tests, respectively. Each spiked sample was prepared by adding 15 (1 st test) or 20 (2nd test) polyethylene particles to 1.0 g of soil that was previously heated at 450 °C for 16 h to eliminate all MP particles that could be present. The extraction and detection of MP in the spiked samples followed all steps described above for the soil samples. The recovery rate was determined by the ratio between the number of identified particles and the number of particles added to each spiked sample. The average and standard deviation of the recovery rates were 80.9% ± 14.3%.

Soil recovery tests were also considered for the manure samples because of the difficulty in obtaining manure samples free of any MP contamination and because the extraction of MP from the manure samples followed a procedure that was similar to the soil samples.

#### Water samples

The recovery test was performed using 4 replicates, each prepared by adding 120 polyethylene particles to a glass beaker containing 200 mL of deionised water. The extraction followed the procedure described above for this type of sample. The average and standard deviation of the recovery rate were 75.0% ± 16.7%.

#### Air deposition samples

For the recovery test, 3 replicates were prepared by adding 20 polyethylene particles to a 1 L glass flask. The extraction procedure followed the method described above. The average and standard deviation of the recovery rate were 96.2% ± 11.1%.

### *Microplasticinput estimation*

Three main sources of MP were identified, i.e. irrigation water, atmospheric deposition and manure. The determination of MP influx from each source is explained below.

#### Water

Input rate (particles·m^−2^·year^−1^) = irrigation rate (m^3^ of water·m^−2^·year^−1^) × concentration of MP in water (particles·m^−3^).

Irrigation rate: 1437.0 m^3^·ha^−1^·year^−1^ = 0.1437 m^3^·m^−2^·year^−1^ (information supplied by the farmers).

The concentration of MP in water was determined directly by our study.

#### Atmospheric deposition

Input rate was determined directly by our study.

#### Manure

Input rate (particles·m^−2^·year^−1^) = application rate (kg of manure·m^−2^·year^−1^) × concentration of MP in manure (particles·kg^−1^).

Application rate = 2500 kg·ha^−1^·year^−1^ = 0.25 kg·m^−2^·year^−1^ (Dias et al., 2017).

Concentration of MP in manure was determined directly by our study.

### Statistical analysis and data visualisation

The statistical analysis and data visualisation were performed using R (version 4.3.1) and R Studio (version 2022.12.0). The normal distribution and homogeneity of variances were tested using Shapiro–Wilk’s and Levene’s tests, respectively, the latter from the “car” package (Fox & Weisberg, 2019). As the data did not follow a normal distribution, differences between groups (i.e. soil layers, MP size categories, and types of samples) were tested using the non-parametric Kruskal–Wallis’ test followed by Dunn’s test. The significance level was set at 0.05. Data visualisation was performed using the “ggplot2” package (Wickham, 2016).

## Results

### MP content in soils

**Fig. 2:**
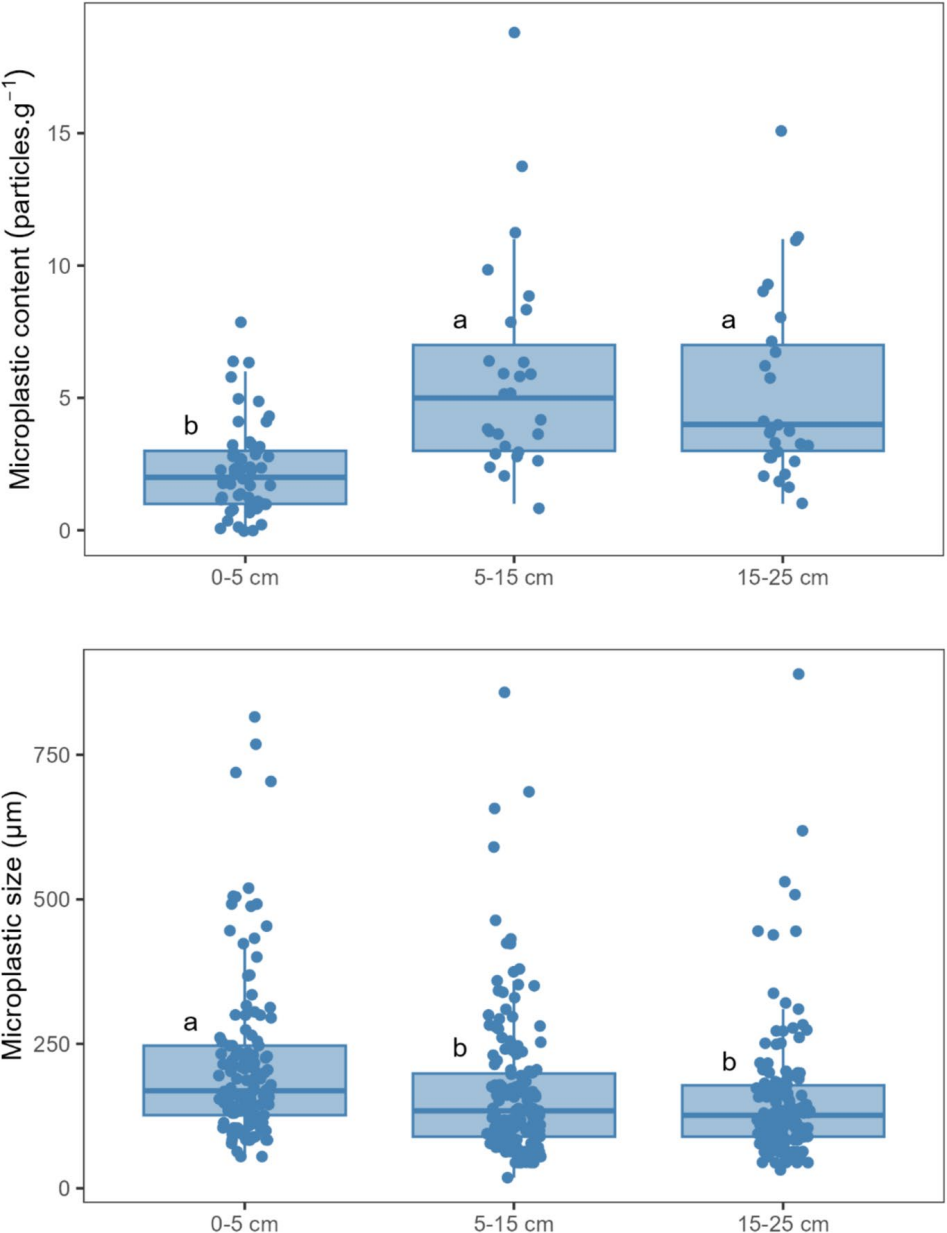
Boxplots showing the microplastic content (top) and the size of microplastic particles (bottom) in the samples collected from 3 soil layers (0–5 cm, 5–15 cm, and 15–25 cm deep). The samples were collected in October 2022, from peach orchards in the east-central Portuguese region of Beira Interior. *N* = 57 samples (0–5 cm layer) and *N* = 27 samples (for 5–15 cm and 15–25 cm layers). In each boxplot, the boxes represent the 1st, 2nd (median), and 3rd quartiles, the whiskers represent 1.5 of the interquartile range or minimum/maximum values, and the dots represent individual sample results. Different letters indicate significant differences between depths determined by Dunn’s test (*p* < 0.05)

**Fig. 3:**
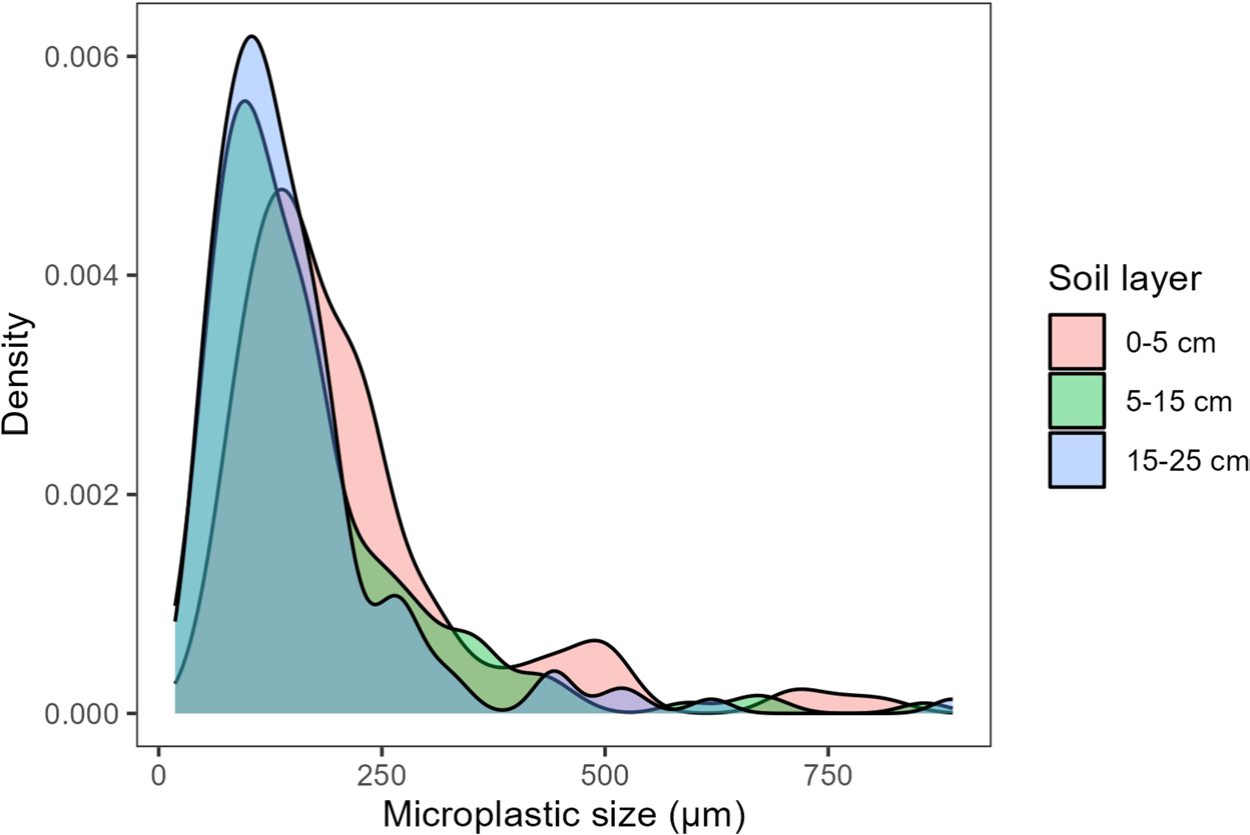
Density plot showing the distribution of microplastics sizes in each soil layer (0–5 cm, 5–15 cm, and 15–25 cm). The samples were collected in October 2022, from peach orchards in the east-central Portuguese region of Beira Interior. *N* = 78 (0–5 cm), *N* = 63 (5–15 cm), and *N* = 54 (15–25 cm)

MP were found in all orchards and in all soil layers that were analysed. MP content varied between 0 (in 5% of the samples) and 8 particles·g^−1^ (average of 2.2 particles·g^−1^) in the 0–5 cm layer, between 1 and 19 particles·g^−1^ (average of 5.9 particles·g^−1^) in the 5–15 cm layer, and between 1 and 15 particles·g^−1^ (average of 5.2 particles·g^−1^) in the 15–25 cm layer (Fig. 2). The size of the particles varied between 55 and 815 µm (average of 217 µm) in the 0–5 cm layer, between 18 and 858 µm (average of 168 µm) in the 5–15 cm layer, and between 32 and 889 µm (average of 155 µm) in the 15–25 cm layer. Statistical analysis indicated a significantly lower (*p* < 0.05) MP content and larger particles in the topsoil layer (0–5 cm) compared to the other 2 deeper layers (Figs. 2 and 3), with most detected particles being below 300 µm (Fig. 3). No statistical differences were found (*p* < 0.05) between layers in the total area of MP particles.

### Potential sources

No statistical differences were found in either the MP concentration or size between the water samples collected upstream and downstream of the dripping tubes (*p* ≥ 0.05; Figure S1, Supplementary material).

**Fig. 4:**
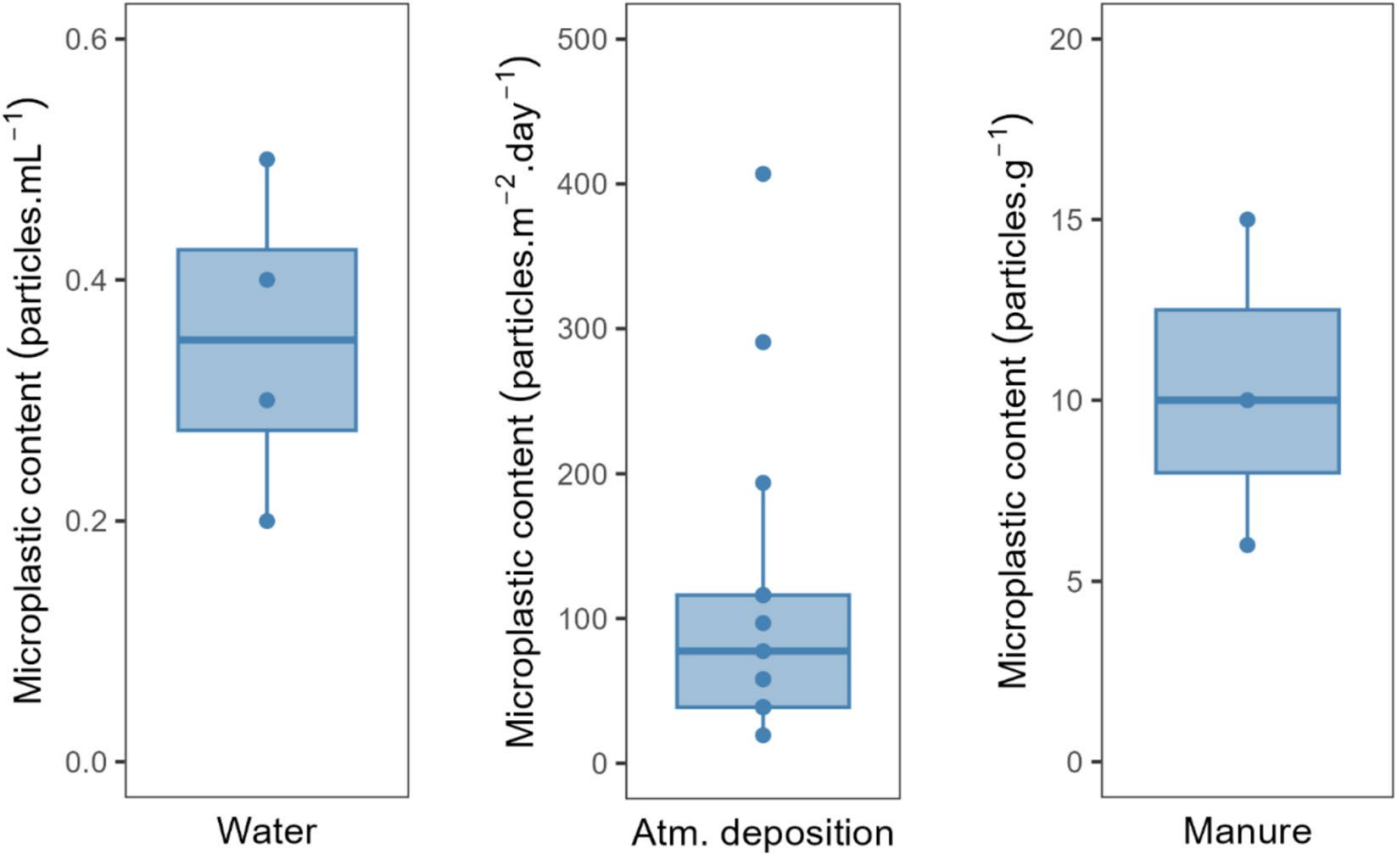
Boxplots representing the input rate of microplastics from water (left), atmospheric deposition (centre), and manure (right) samples collected from peach orchards in the east-central Portuguese region of Beira Interior. Water samples were collected directly under the dripping system. *N* = 4 (water), *N* = 15 (atmospheric deposition), and *N* = 3 (manure). In each boxplot, the boxes represent the 1st, 2nd (median), and 3rd quartiles, the whiskers represent 1.5 of the inter-quartile range or minimum/maximum, and the dots represent the individual results

The MP concentration/content in water, atmospheric deposition and manure samples (Fig. 4) varied between 0.2 and 0.5 particles·mL^−1^ (average of 0.4 particles·mL^−1^), 19.4 and 406.9 particles·m^−2^·day^−1^ (average of 113.7 particles·m^−2^·day^−1^), and between 6.0 and 15.0 particles·g^−1^ (average of 10.3 particles·g^−1^), respectively. These 3 main sources of MP to soils were responsible for the input of 1.02 × 10^5^ particles·m^−2^·year^−1^. Water was the main contributor, transporting 55.9% of the particles, atmospheric deposition was the second main contributor (41.2%), and manure was the third (2.9%).

**Fig. 5:**
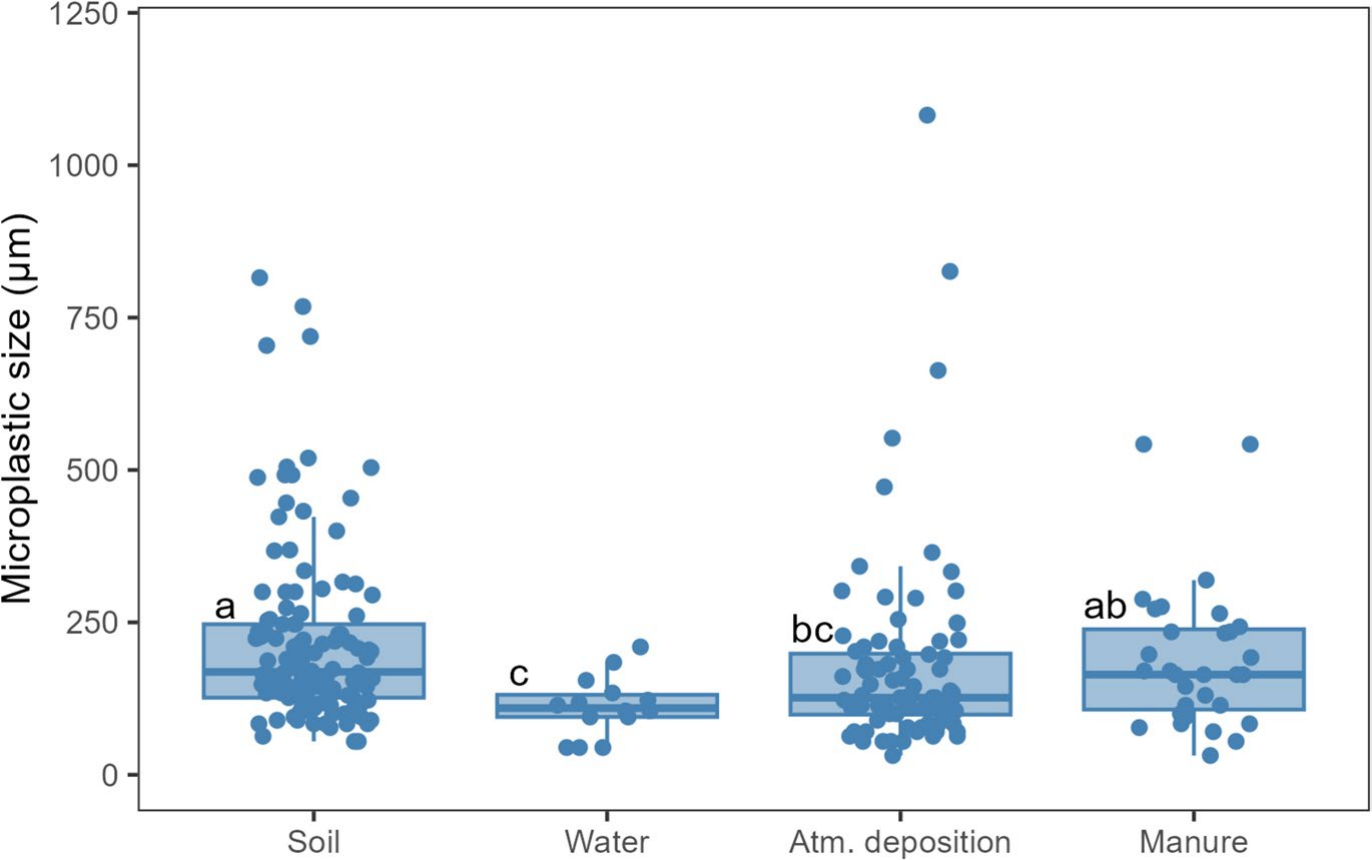
Boxplots representing the comparison of microplastic sizes in soil (0–5 cm layer) and its 3 main potential sources of contamination, i.e. water (collected directly from the dripping system), atmospheric deposition, and manure. *N* = 128 particles (soil), *N* = 14 particles (water), *N* = 88 particles (atmospheric deposition), and *N* = 31 particles (manure). Samples were collected from peach orchards in the east-central Portuguese region of Beira Interior. Different letters indicate significant differences (*p* < 0.05) determined by Dunn’s test. In each boxplot, the boxes represent the 1st, 2nd (median), and 3rd quartiles, the whiskers represent 1.5 of the inter-quartile range or minimum/maximum, and the dots represent individual results

**Fig. 6:**
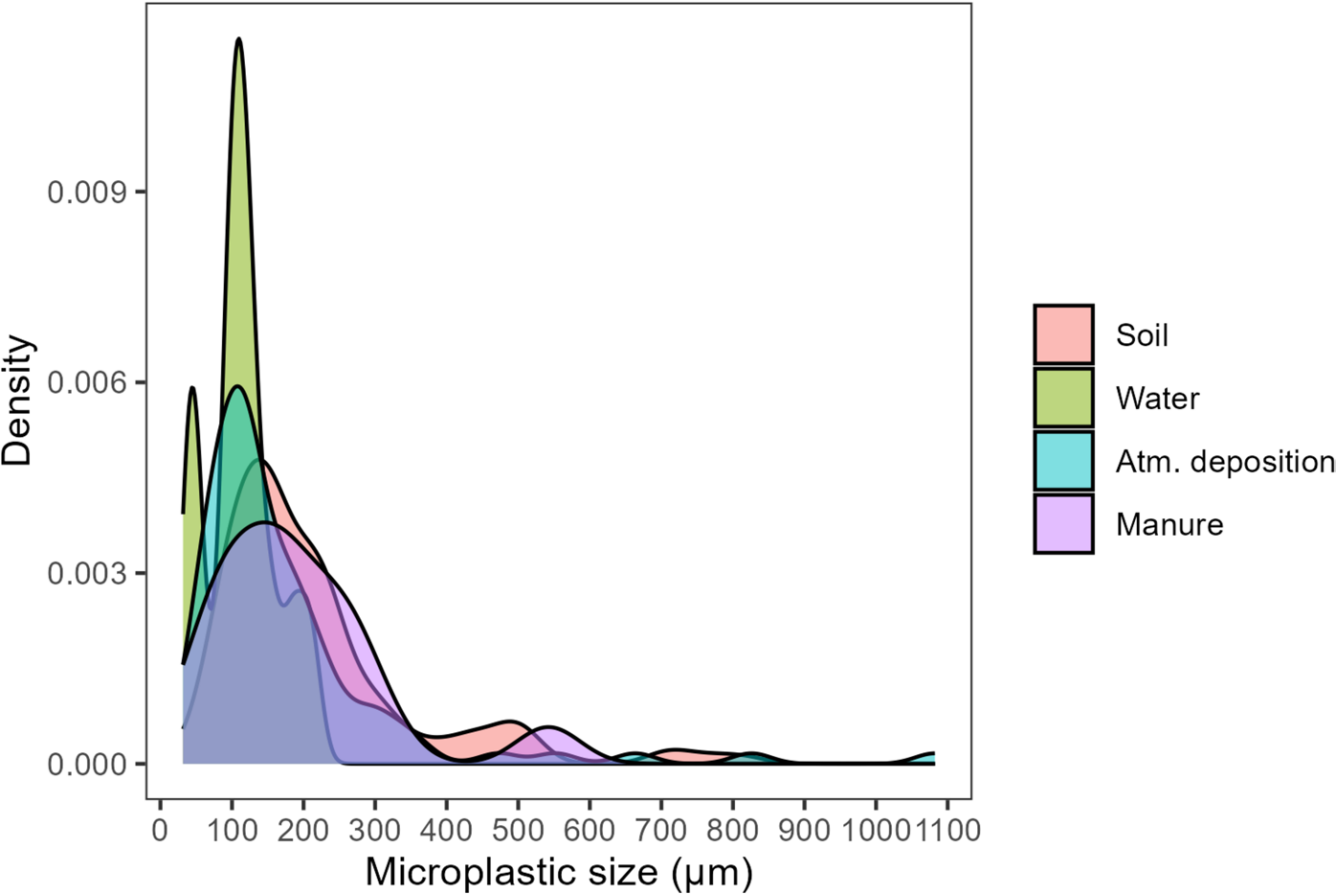
Density plot representing the distribution of microplastic sizes in soil (0–5 cm layer) and in its 3 main potential sources of contamination, i.e. water (collected directly from the dripping system), atmospheric deposition, and manure. *N* = 128 (soil), *N* = 14 (water), *N* = 88 (atmospheric deposition), and *N* = 31 (manure). Samples were collected from peach orchards in the east-central Portuguese region of Beira Interior

The size of the particles (Figs. 5 and 6) ranged between 44.7 and 209.8 µm (average of 112.3 µm) in water samples taken from drippers, between 31.6 and 1082.1 µm (average of 177.6 µm) in atmospheric deposition samples and between 31.6 and 542.2 µm (average of 191.5 µm) in manure samples. Most of the detected particles were below 300 µm. The MP particles collected from soil samples were significantly larger (*p* < 0.05) than the particles collected from water and atmospheric deposition samples.

## Discussion

### Soil contamination

This study determined the MP content in soils under peach orchards from the east-central Portuguese region of Beira Interior. Additionally, it evaluated the relevancy of 3 main potential sources of MP to the soil: irrigation water, atmospheric deposition, and manure. It also tested the hypothesis that irrigation pipes contribute to an increase in MP concentration in irrigation water, which was not statistically proven.

A statistically significantly higher MP content was found in the soil layers 5–15 cm (average of 5.9 particles·g^−1^) and 15–25 cm (average of 5.2 particles·g^−1^) compared to the shallower layer, i.e. 0–5 cm (average of 2.2 particles·g^−1^). Furthermore, the average size of the particles found in the deeper layers (5–15 cm and 15–25 cm; average of 168 µm and 155 µm, respectively) was smaller than in the shallower layer (0–5 cm, average of 217 µm). In this regard, the results found in the literature are contradictory. For example, Wang et al. (2023), like our study, reported higher MP content in the deeper layer, whilst Zhang et al. (2022) found the opposite.

Comparisons between studies should be approached with caution. Firstly, the depth of the analysed soil layers differs across studies. For example, in our study, we analysed the 0–5 cm, 5–15 cm, and 15–25 cm soil layers, whilst Wang et al. (2023) analysed the 0–20 cm, 20–40 cm, and 40–60 cm layers, and Zhang et al. (2022) analysed the 0–20 cm and 20–40 cm layers. Soil type, texture, and management activities, such as tillage, may promote the movement of plastic particles in soil (H. Zhang et al., 2022). This did not occur in our study because soil tillage is not a common practice in the peach orchards from which the samples were collected. The types of MP present in soil are also likely to be different across fields, regions, and studies. Later sampling campaigns, like ours, are likely preceded by surface runoff events which can affect plastic load on soil surface via transport of particles within or between fields (J.-J. Guo et al., 2020; Lwanga et al., 2022).

### Potential sources and their relative importance

We identified 3 main sources of MP particles to the soil under the studied orchards: water, atmospheric deposition, and manure.

Similarly to our results, irrigation water was already identified as an important source of MP to soil by other authors (S. Guo et al., 2023; Jiang et al., 2023; Lwanga et al., 2022). This is especially applicable to our study as the MP content is generally higher than what was presented by other authors who worked with water samples collected from irrigation structures, rivers or aquifers (Gong et al., 2023; Ismanto et al., 2023; Jiang et al., 2023; Napper et al., 2023; W. Zhao et al., 2024; Zhou et al., 2020).

In our study, irrigation pipelines, which are made from polyethylene, are also a potential source of MP to soil either directly from their surfaces, which are exposed to sunlight, or from their inner side, which may be adding particles to the water circulating inside. The latter hypothesis was tested by us and, although the statistical results did not confirm it at a confidence level of 95%, they did not allow us to discard it. The first hypothesis was not tested in our study. Nevertheless, the exposure of the plastic tubes to the sunlight can contribute to the degradation of their surfaces, and therefore, the release of MP to the ground is expected (J.-J. Guo et al., 2020; Lwanga et al., 2022; Napper & Thompson, 2023).

The role of wind in the transport of MP has already been studied and proven by other studies (Bullard et al., 2021; Rezaei et al., 2019, 2022). Dry regions prone to desertification, like the Portuguese east-central region of Beira Interior where our study area is located, are sensitive to this type of transport (Lwanga et al., 2022; Nunes et al., 2023). On the one hand, the bare soil resulting from herbicide application is a factor that potentially enhances wind erosion and, therefore, the removal of some MP from the soil surface (Lwanga et al., 2022; Nunes et al., 2023; Rezaei et al., 2022). On the other hand, the trees may contribute to slow down the wind, capturing some of the particles (including MP) transported by it (Asensio-Amador et al., 2022). The MP content in air deposition samples was of the same order of magnitude as what was found by Napper et al. (2023) but lower than what was reported by Rezaei et al. (2022). Different methodologies used for sampling and analysis should not be discarded as a reason for differences amongst the results from different studies. Nevertheless, it should be noted that wind can not only add MP to the soil but also remove them.

The manure applied in the orchards followed in our study comes from sheep herds and was identified as the least important source of MP to the soil amongst the 3 potential sources evaluated in our study. Other studies have already identified this organic soil amendment as an important source of MP (R.-T. Wu et al., 2021; Yang et al., 2021; J. Zhang et al., 2023). The ingestion of plastic debris and MP particles by sheep is an important reason for its appearance in manure (Beriot et al., 2021). Nevertheless, bale nets and strings used to collect and store hay and other sheep feed can also be sources of particles to the animal beds and, therefore, to the manure (Lwanga et al., 2022).

The average value for the MP content in the 0–5 cm soil layer was 2.2 particles·g^−1^, which, considering 1.5 g·cm^−3^ as the reference value for soil bulk density, results in 1.7 × 10^5^ particles·m^−2^. The 3 identified sources were responsible for an average of 1.02 × 10^5^ particles·m^−2^·year^−1^. Therefore, if input rates remain the same, the average value of MP content in the 0–5 cm soil layer can double in less than 2 years. There are, of course, pathways and technical aspects that can lower the number of particles in the surface soil layer, namely the vertical transport of particles into deeper layers, wind erosion, and fragmentation into smaller particles below the detection limit (J.-J. Guo et al., 2020; Lwanga et al., 2022; Napper & Thompson, 2023). The decrease in size that follows this fragmentation eventually produces nanoplastics and facilitates the uptake of particles by living organisms, including plants and soil biota. Some studies suggest that plastic degradation and mineralisation by living organisms are possible which may play a role in the remediation of contaminated sites. However, this uptake often results in bioaccumulation in the bodies of those organisms and in biomagnification, i.e. in an increase of micro and nanoplastic contents throughout food chains. The consequences of this are complex because the materials and additives present in plastic particles are very diverse and because these particles may interact with other pollutants. In any case, they are usually harmful to individual organisms and, consequently, to entire ecosystems as well (Arif et al., 2024; De Souza Machado et al., 2018; Lwanga et al., 2022; Thapliyal et al., 2024). Despite the risks that MP pose to ecosystems, there is still an extensive absence of comprehensive EU legislation applicable to MP (European Commission, 2024b).

### Limitations, strengths and suggestions for future research

The extraction and quantification of MP used in this study were based on differences between their density and the density of other particles present in the sample, as well as their behaviour when heated. These methods are capable of extracting and identifying MP whilst being relatively inexpensive and fast, allowing the analysis of a higher number of samples and thereby increasing the representativeness of the study compared to more expensive, time-consuming methods (e.g. high density separations and IR imaging techniques) (Beriot et al., 2021; Leitão et al., 2023). Despite these advantages, the methods used in this study also have limitations, which we discuss below.

Water was used as the extracting medium for soil samples, limiting the extraction to particles with densities lower than 1.0 g·cm^−3^. An alternative would be using a salt solution, whose density can reach 1.4–1.5 g·cm^−3^, allowing the extraction of particles with densities up to that limit. Nevertheless, it would also extract more organic matter particles, requiring a digestion step to be added. Digestion treatments may also affect MP, leading to their partial degradation or fragmentation and reducing overall extraction recovery. The multitude of plastic polymers and their additives makes it virtually impossible to accurately predict the effect of organic matter digestion on plastic particles (Bläsing & Amelung, 2018; He et al., 2018). For manure samples, the organic matter digestion was unavoidable due to its high content. We have chosen the method that potentially results in the least degradation of plastic particles (He et al., 2018).

As explained above, the identification of MP in this study was based on the change of shape of the plastic particles when heated at 150 °C for 30 s. The heating temperature and time are a compromise: increasing temperature or heating time could result in more plastic particles changing shape due to the heating, but it could also result in the complete combustion of more particles, which would not be detected. The method used for MP extraction in water and atmospheric deposition samples was different due to the nature of the samples and did not require an extracting medium or organic matter digestion, explaining the much higher recovery results.

The method validation was carried out using a limited number of polymers or plastic materials, much lower than the diversity of materials that can potentially be present in the samples. Different methods will also result in different limits of detection/quantification, affecting the number of MP identified.

The visual identification method is adequate to identify MP, as shown by the results from the validation tests performed within this work and other studies (Beriot et al., 2021; Leitão et al., 2023; S. Zhang et al., 2018). However, it does not allow the identification of the polymers present in the samples. This analysis, although being time-consuming and expensive, should be addressed in future works to better characterise soil contamination in MP and its main sources.

Plastic litter (e.g. plastic bottles, strings, and packages) was not commonly seen on the soil of the orchards. However, it may exist and contribute to soil contamination in MP. The importance of this source should be addressed in future studies.

Sewage sludge is referred in other studies as a major source of MP to the soil (Corradini et al., 2019; Lwanga et al., 2022). After asking the farmers and the farmers’ associations, we learned that sewage sludge was not applied to the orchards in, at least, the last 2 years. However, older applications are possible in some orchards and might have contributed to soil MP contamination.

A higher content of smaller particles was found in the deeper soil layers analysed in this study (5–15 cm and 15–25 cm), indicating a migration of MP deeper into soil and the potential to reach aquifers. Therefore, it is important to further investigate the fate and impacts of different types of plastic debris. Additionally, irrigation water was found to be a major source of MP to the soil under the studied orchards. Our study on the role of irrigation tubes in the water contamination by MP was inconclusive. Moreover, the ponds or river dams from where the water is sourced may also have a significant MP content contributing to the MP contamination of the water used for irrigating the orchards. Future studies should clarify the origin of MP contamination in irrigation water.

The need to prevent MP contamination has been recognised by the EU, even though comprehensive laws applying to MP are still lacking despite their importance in achieving the goal of having all soils in healthy conditions by 2050. Within this framework, the crop and region focused on by this study can be taken as a case study whose results can potentially be applicable in other situations, especially within agricultural contexts. The management of peach orchards is similar to the management of other crops, especially perennials, and similar edaphoclimatic conditions can be found in other regions, particularly those around the Mediterranean.

MP pollution is a widespread problem affecting several environmental compartments, including soil. Despite this, the available information regarding potential sources of MP to agricultural soils, although highly relevant, is still limited, especially with regard to permanent crops in the aforementioned region. Our results help to reduce this knowledge gap by providing more information regarding MP contamination of agricultural soils and the importance of its potential sources, which may contribute for a better understanding of this subject. In addition to its scientific relevancy, this knowledge is important to raise awareness on stakeholders, including farmers, plastic manufacturers, and policy makers, regarding the importance of an integrated approach that results in preventive measures that help, if not to avoid, at least to decrease MP spreading into the environment.

## Conclusions

This study, focusing on MP contamination in soil and its main sources, corroborates high and widespread MP contamination in agricultural soils. More studies are needed to better understand the dynamics related to MP in soil and the main polymer types that are present. Irrigation water was identified as one of the major MP sources to soil, stressing the need to safeguard the quality of the water bodies from which the irrigation water is sourced, and of water bodies in general. Our results indicate a vertical movement of MP into deeper soil layers, raising serious concerns about the possible contamination of aquifers that are, in some cases, used for human consumption. It is suggested that future works should extend the survey presented in our study to other crops and regions, characterise the main polymers responsible for the contamination and evaluate possible temporal variations in MP contamination. Considering the negative impact of MP in soil ecosystems, this knowledge is essential to accurately select adequate measures that may prevent and ameliorate this type of soil contamination and help to achieve the EU goal of having all soils in healthy condition by 2050.

## Supplementary Information

Below is the link to the electronic supplementary material.

Supplementary file1 (PDF 142 KB)

## Data Availability

Data will be made available on request.
