# Supplementary material for: Tracking the source of microplastics in soil—an exploratory case study in peach orchards from east-central Portugal
Source: Environ Monit Assess. 2025 May 10;197(6):645. doi: 10.1007/s10661-025-14072-9 (PMC12065684; doi:10.1007/s10661-025-14072-9)
Supplement: Supplementary file 1 — Supplementary file1 (PDF 142 KB) [file 10661_2025_14072_MOESM1_ESM.pdf]

Supplementary material

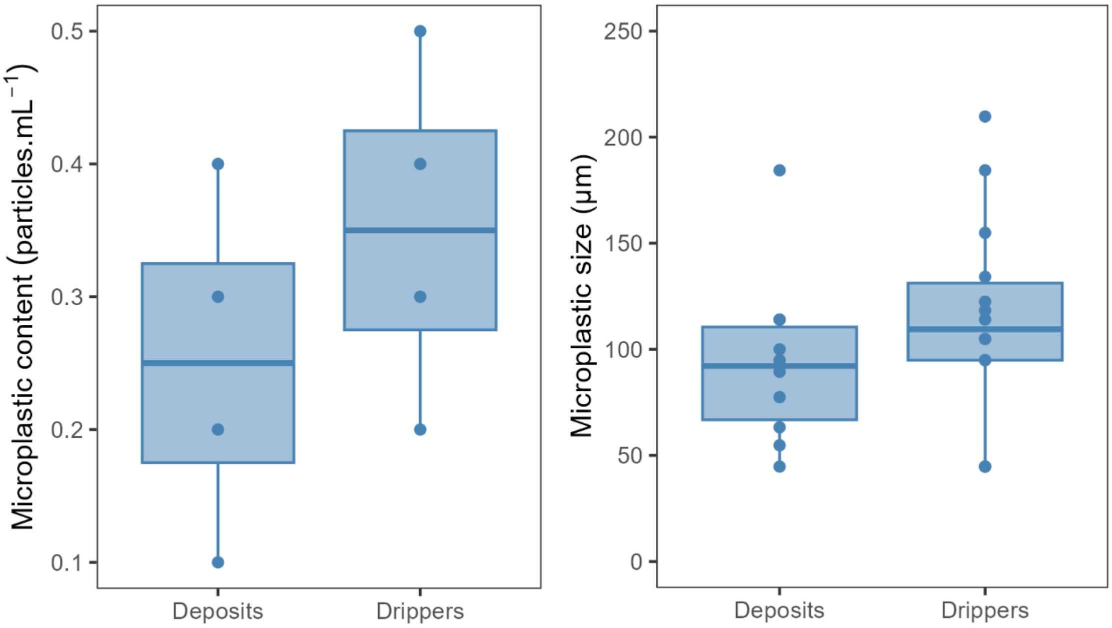

Figure S1. Boxplots representing the microplastic content (left), and the microplastic size (right) for the samples collected upstream to the dripline and directly from the drippers. In each boxplot, the boxes represent the 1st, 2nd (median), and 3rd quartiles, the whiskers represent 1.5 of the inter-quartile range or minimum/maximum, and the dots represent individual results.
